# Comparative Appraisal of Intravascular Ultrasound and Optical Coherence Tomography in Invasive Coronary Imaging: 2022 Update

**DOI:** 10.3390/jcm11144055

**Published:** 2022-07-13

**Authors:** Piotr Baruś, Jakub Modrzewski, Karolina Gumiężna, Piotr Dunaj, Marcin Głód, Adrian Bednarek, Wojciech Wańha, Tomasz Roleder, Janusz Kochman, Mariusz Tomaniak

**Affiliations:** 1 First Department of Cardiology, Medical University of Warsaw, 02-091 Warsaw, Poland; piotrbarus@op.pl (P.B.); kuba.modrzewski@gmail.com (J.M.); kgumiezna@gmail.com (K.G.); piotr.dunaj@wum.edu.pl (P.D.); marcinglod5729@gmail.com (M.G.); adikbednarek@gmail.com (A.B.); janusz.kochman@wum.edu.pl (J.K.); 2 Department of Cardiology and Structural Heart Diseases, Medical University of Silesia, 40-055 Katowice, Poland; wwanha@sum.edu.pl; 3 Department of Cardiology, Regional Specialist Hospital in Wrocław, 51-124 Wroclaw, Poland; tomaszroleder@gmail.com

**Keywords:** intravascular imaging, intravascular ultrasound, optical coherence tomography, percutaneous coronary interventions

## Abstract

Although coronary angiography has been well established as a standard modality for percutaneous coronary intervention guidance, recent developments in intravascular imaging techniques, such as intravascular ultrasound and optical coherence tomography, have become increasingly adopted, enabling direct detailed lesion visualization, including lesions beyond the scope of assessment using exclusively angiography. Intravascular imaging modalities have been reported to potentially improve both short- and long-term percutaneous intervention outcomes. This review aims to provide a comparative summary of recent advancements in research regarding the clinical applications and outcomes of intravascular ultrasound and optical coherence tomography.

## 1. Introduction

The last three decades have brought advancements in intravascular imaging technologies, e.g., intravascular ultrasound (IVUS) and later optical coherence tomography (OCT) were introduced and developed to gradually play an increasingly essential role in guiding percutaneous coronary interventions (PCI), along with the critically acclaimed coronary angiography (CAG). Because limitations of angiography are widely acknowledged, increasing attention is brought to intravascular imaging. It makes direct visualization of the vascular wall feasible and enables more precise evaluation of plaque burden, plaque composition and vessel remodeling. Intravascular imaging provides interventionalists with invaluable help in multiple ways by informing on the necessity of lesion preparation and stent sizing, guiding stent expansion, identifying acute complications or assessing late stent failure [1]. Both IVUS and OCT are recognized to contribute to positive clinical outcomes when used complementarily to CAG in coronary artery disease assessment and guiding PCI [2,3,4,5]. Moreover, a retrospective cohort study from the National Inpatient Sample (2004–2014) showed that both modalities are used more frequently, with a 22-fold increase in IVUS and 118-fold increase in OCT in a 10-year time period [6]. The current guidelines of the European Society of Cardiology recommend both IVUS and OCT as methods of choice for the diagnosis of spontaneous coronary artery dissection if the coronary angiogram is unclear regarding this matter [7,8]. Moreover, both modalities are recognized by the guidelines in deepening the diagnostic process in case of a myocardial infarction with non-obstructive coronary arteries [9,10]. The 2018 ESC guidelines for revascularization define IVUS as a useful tool for planning a therapeutic invasive strategy among patients with intermediate grade left main coronary artery stenosis [11]. IVUS should be taken into consideration when analyzing the severity of lesions in the unprotected left main (class IIA, level B). Not only IVUS, but also OCT can be used for identifying stent restenosis (class IIA, level C). Both modalities are also described in the guidelines as methods for PCI optimization (class IIA, level B) [12]. Nevertheless, each technique shows a distinct set of capabilities regarding various clinical applications, which are compared and contrasted below based upon the available evidence.

## 2. Advantages and Disadvantages of IVUS Imaging

**Figure 1:**
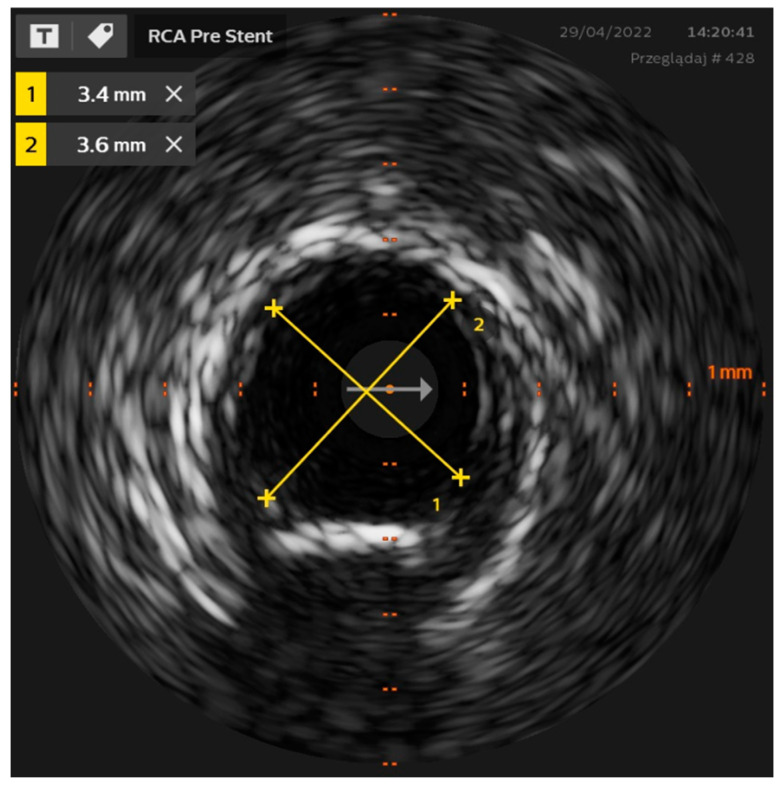
IVUS image of the right coronary artery—diameters’ measurements.

Intravascular ultrasound (IVUS) is the first catheter-based technology used for intravascular imaging, introduced by Yock et al. in the 1980s [13]. In principle, IVUS depends on a miniaturized piezoelectric transducer mounted on the catheter tip that emits ultrasonic waves (at 20–60 MHz) [1,14]. The ultrasounds are emitted radially either by mechanical rotation of a single transducer or a sequentially activated array of fixed transducers, depending on the catheter design type [15]. As the amplitude of the backscattered waves and the echo time delay are processed, a series of cross-sectional grey-scale images are obtained (Figure 1).

A major advantage of IVUS stems from its relatively deep tissue penetration (5–6 mm) [1] (Table 1). This allows full-thickness visualization of the vessel wall, enabling the usage of vessel size parameters, such as the external elastic membrane (EEM) diameter or reference lumen diameters for stent sizing [16], which plays a significant role in PCI optimization, as overestimation or underestimation of stent size may instigate complications such as coronary dissection, perforation, extensive malapposition or stent underexpansion [17]. Additionally, visualizing all layers of the vessel wall can be useful in follow-up assessment, being able to show vessel remodeling processes [18] or providing detailed imaging when angiography suggests late acquired stent malapposition (persistent staining or aneurysmal change) [1]. Importantly, IVUS is the only imaging tool, which enables to provide plaque burden.

However, IVUS has a relatively low resolution. Its axial resolution is 100–150 μm and lateral 150–300 μm for 40 MHz, whereas for 60 MHz, it ranges between 40–60 μm and 60–140 for axial and lateral, respectively [14,17]. It may be a limiting factor when more detailed evaluation is needed, e.g., superficial plaque assessment or suboptimal PCI result identification (coronary dissections, underexpansion, tissue protrusions, etc.) [19]. Acknowledging the limitations of qualitative visual interpretation of grey-scale IVUS images, several post-processing methods have been developed to augment coronary plaque tissue characterization, such as VH-IVUS (virtual histology), iMAP-IVUS (iMap-Intravascular Ultrasound Radiofrequency Signal Analysis) or IB-IVUS (integrated backscatter). VH-IVUS deploys autoregression models to analyze underlying frequency content of the reflected radiofrequency signals enabling for classification of plaque tissue components into colorful images [15,20]. Studies have shown that raw backscattered frequency analysis (IVUS-RF) can be useful in plaque characteristics assessment in high-risk populations, possibly allowing to stratify cardiovascular risk, along with clinical and angiographic findings [18,21].

Another advantage of the IVUS method lies in its independence from the need for contrast injections and blood clearance from the vessel, which was found to be able to reduce the total amount of contrast compared to angiography-guided PCI [22]. It also makes IVUS the preferred modality in patients with renal failure and in ostial left-main lesion assessment and guidance, as in these settings, blood clearance may be challenging [2].

IVUS shows limitations when it comes to the assessment of calcified plaque, as calcium scatters most of the ultrasound signal, making evaluation of plaque behind it not feasible [17]. However, IVUS was shown to be able to detect angiographically invisible calcium [23]. On the other hand, IVUS is useful in assessment of lipid-rich plaque and red thrombotic structures [24]. One other extension of classic IVUS is its combination with the near-infrared spectroscopy (NIRS-IVUS), which allows to differentiate between lipid and non-lipid plaques [25,26].

In the ULTIMATE trial, a multicenter prospective randomized study, Zhang et al. enrolled a total of 1448 patients who required DES implantation. They were randomly assigned to undergo PCI in either an IVUS-guided or angiography-only-guided group. After 12 months, the incidence of target vessel failure (TVF) was assessed, including cardiac death, target-vessel myocardial infarction and clinically driven target-vessel revascularization. At 12 months follow-up, 60 TVFs (4.2%) occurred, with 21 (2.9%) in the IVUS group and 39 (5.4%) in the angiography group (*p* = 0.019). Hence, a statistically significant reduction in target vessel failure rate was shown in the IVUS-guided group, which the authors mainly ascribe to IVUS being critical in complex lesion assessment, guiding post-dilation and minimizing or edge complications during PCI. There is evidence to suggest that IVUS-guided DES implantation significantly improved clinical outcomes in all-comers, particularly for patients who had an IVUS-defined optimal procedure, compared with angiography guidance [27].

## 3. Advantages and Disadvantages of OCT Imaging

OCT is an intravascular imaging modality that uses infrared light (1–3 μm wavelength), which provides very high spatial resolution (axial 10–20 μm and lateral 20–90 μm), approximately ten times greater than that of IVUS [17]. Thus, OCT enables a more detailed evaluation of the superficial plaque and of the endoluminal surface of the vessel (e.g., TCFA, stent architecture or plaque rupture) [17,28]. OCT might also allow detection of small, subtle abnormalities of as of yet undetermined clinical significance [24].

Despite increased imaging resolution of OCT, the method also carries limitations. Since the infrared light (wavelength of 1250–1350 nm) does not penetrate through hemoglobin, there is a need for complete blood clearance from the vessel using the contrast agent [1]. For the same reasons, OCT is not always feasible in the assessment of ostial left main coronary artery lesions [2] and in patients with severe renal failure. However, recently, several different flushing agents were investigated, including crystalloids such as Ringer’s solution or normal saline. Saline has been shown to produce images with similar quality to contrast OCT acquisitions. Nevertheless, difficulties with blood mixing and potential risk of triggering arrhythmia with non-contrast flushes exist. Alternative non-contrast flush media with similar biocompatibility, viscosity, blood clearance capability, and optical transparency are being investigated [3].

OCT has a significantly lower tissue penetration depth (1–2.5 mm) [2] (Table 1), leading to limited visualization of the entire vessel wall. Thus, the plaque burden estimation is impossible by OCT. On the other hand, OCT identifies plaque composition with high accuracy, and is the gold standard to detect vulnerable plaques in vivo [29,30,31].

Furthermore, OCT provides high resolution images of the implanted stent is extremely useful to guide PCI. However, lumen values obtained by OCT are lower than those provided by IVUS. Therefore, novel OCT-guided stent sizing optimization algorithms [19] are based on proximal and distal segment reference EEL measurements [1,24]. In addition, due to superior resolution, the reproducibility of OCT measurements is better than IVUS [1].

**Figure 2:**
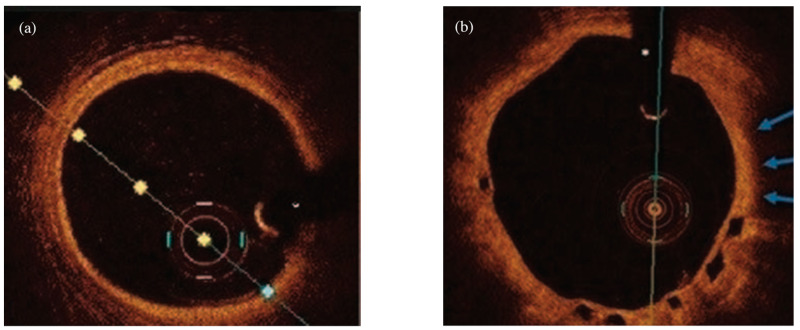
Examples of optical coherence tomography imaging: (**a**) demonstrating normal artery wall comprising three-layered architecture: highly backscattering thin layer-intima, low backscattering-media and heterogeneous layer-adventitia (**b**) an example of thin-cap fibroatheroma (blue arrows) visible in one-year follow-up after bioresorbable vascular scaffold implantation.

The assessment of the coronary artery pre-PCI consists of a thorough analysis, where OCT can visualize: the culprit lesion responsible for the myocardial infarction, morphology of the lesion, which is a decisive factor in further therapeutic decisions regarding stent length, diameter and its landing zone. All of the above are mentioned in the MLD MAX algorithm (Morphology, Length, Diameter, Medial dissection, Apposition, Expansion). This deepened evaluation optimizes PCI results and clinical outcomes of patients. However, in order to benefit from intracoronary imaging modalities, it is important to understand the layered anatomy of the vessel wall and the acquired OCT or IVUS images. A healthy coronary artery wall comprises three layers (Figure 2); a lack of such a structure is an indication of pathology, including the most common abnormalities: fibrous plaque, high lipid plaque, calcified lesions or thrombi. If a fibrous plaque is visualized, a balloon pre-dilatation or direct stenting is recommended. On the other hand calcified plaque requires pre-dilatation with a non-compliant (NC) balloon, the use of a cutting balloon or intracoronary lithotripsy [3].

Another strength of OCT is that infrared light may penetrate some calcium deposits, providing detailed and spatial representation of their morphology, and thus, making OCT a preferred modality for calcified plaque assessment [17]. A fibrocalcific plaque is described as a heterogeneous lesion in the vessel wall. The OCT scan shows fibrocalcific plaques as well-delineated signal-poor regions with sharp borders, which are typically surrounded by brightly visualized fibrous tissue. It makes OCT useful in the decision-making process to deploy techniques used to treat heavily calcified lesions, such as rotational/orbital atherectomy or excimer laser coronary atherectomy. It enables the visualization of the entire calcified lesion and allows to determine its localization, thickness, distribution and volume using three-dimensional reconstructions, which aids in the choice of the most suitable atherectomy technique [3].

Recently, an OCT scoring system was developed to predict the risk of stent underexpansion [32]. The software uses OCT pullbacks to assess the anatomy of coronary arteries, supported by artificial intelligence, which enables quick calcification quantification and vessel sizing, therefore contributing to stent optimization.

Another advantage of OCT imaging is its suitability for three-dimensional (3D) reconstruction, facilitated by its high resolution, which provides accurate volumetric images of the coronary tree. It might be useful in specific situation such as stent fracture [17] or assessment of lesions located in coronary artery bifurcations.

## 4. Direct Comparison

Thus far presented evidence can lead to a conclusion that both modalities are complementary to each other; OCT is more suitable for plaque morphology assessment, whereas IVUS visualizes the media and adventitia [33]. Many studies were conducted to compare both techniques in certain situations (Table 2). It was proven that both IVUS and OCT can detect diffuse intimal and medial thickening and luminal narrowing at the spasm site of the coronary artery [34,35]. However, there is evidence that OCT enables more accurate detection of functionally significant intermediate non-left main coronary artery stenoses than intravascular ultrasound [36]. A meta-analysis of 7537 lesions in 6919 patients showed that IVUS- and OCT-derived minimum lumen area (MLA) had a similar sensitivity in predicting hemodynamically significant lesions (IVUS-MLA: 0.747 vs. OCT-MLA 0.732, *p* = 0.519). However, OCT-MLA had a higher specificity (0.763 vs. 0.665, *p* < 0.001) and diagnostic accuracy in detecting flow-limiting stenoses than IVUS-MLA (AUC 0.810 vs. 0.754, *p* = 0.045).

In the assessment of calcified lesions, OCT has an advantage over IVUS due to its capacity to visualize and measure calcium thickness. This is a result of the different waves used in each modality. Ultrasound waves nearly fully reflect from calcified lesion, whereas infrared waves are able to penetrate calcium [3].

**Figure 3:**
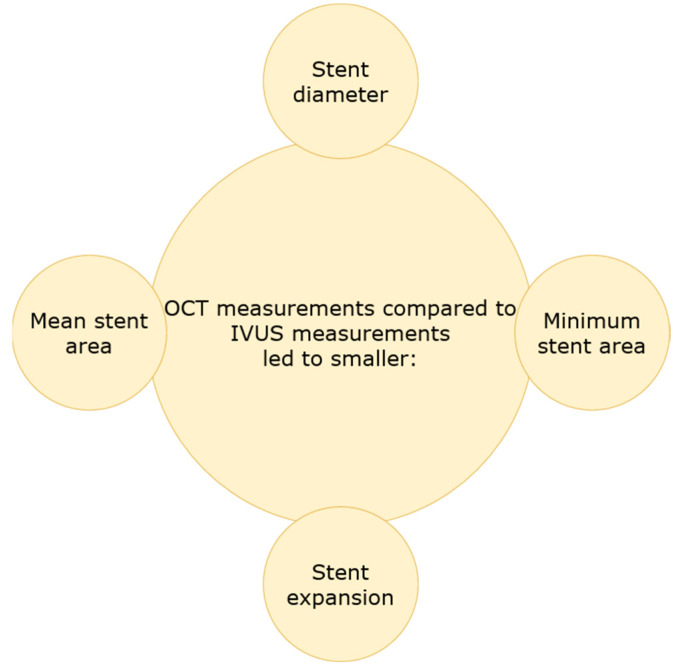
OCT vs. IVUS measurement outcomes.

Several studies sought to investigate the differences of both modalities in terms of stent implantation and how they can be used to optimize PCI. Habara et al. examined 70 patients with stable and unstable angina with FD-OCT and IVUS, in which stents were implanted. The visibility of the vessel border was significantly lower in the OCT group (*p* < 0.05). Moreover, the minimum and mean stent area, focal and diffuse stent expansion were smaller in the OCT group (6.1 ± 2.2 mm vs. 7.1 ± 2.1 mm, 7.5 ± 2.5 vs. 8.7 ± 2.4 mm, 64.7 ± 13.7% vs. 80.3 ± 13.4%, 84.2 ± 15.8% vs. 98.8 ± 16.5%, *p* < 0.05, respectively). The frequency of significant residual reference segment stenosis at the proximal edge was higher in the OCT group (*p* < 0.05). There were no significant differences in pre-balloon dilatation and stent size; however, the deployment pressure was higher in the IVUS group. In addition, in the IVUS group, there was observed less residual stent-edge plaque burden. Therefore, this study concluded with smaller stent expansion and more frequent significant residual reference segment stenosis in the OCT-guided stent implantation in comparison to the IVUS-guided group [37]. Kubo et al., in the prospective OPINION study, randomly allocated 829 patients to perform IVUS-guided or OCT-guided PCI. The stent diameter was smaller in the OCT group (*p* = 0.005), and there was a tendency towards longer stents in the OCT-guided PCI (*p* = 0.06) [38]. The ILUMIEN III: OPTIMIZE PCI randomized controlled trial enrolled patients from 29 hospitals in 8 countries, who presented with one or more lesions located in a native coronary artery; however, left main, ostial right coronary artery stenoses, bypass graft stenoses, chronic total occlusions, planned two-stent bifurcations and in-stent restenoses were excluded. In total, 450 patients were divided into groups with OCT, IVUS or angiography-guided stent implantation. All patients underwent final OCT imaging. The final median minimum stent area was 5.79 mm^2^ (IQR 4.54–7.34) with OCT guidance, 5.89 mm^2^ (4.67–7.80) with IVUS guidance and 5.49 mm^2^ (4.39–6.59) with angiography guidance. OCT guidance was non-inferior to IVUS guidance (one-sided 97.5% lower CI −0.70 mm^2^; *p* = 0.001), but not superior (*p* = 0.42). OCT guidance was also found not to be superior to angiography guidance (*p* = 0.12) [19]. The studies presented above have shown that OCT, in comparison with IVUS, gives smaller measurement of stent diameter, minimum stent area, mean stent area, focal and diffuse stent expansion (Figure 3). This finding can be presumed to stem from the deeper tissue penetration provided by IVUS. However, clinical significance of these observation is yet to be determined.

Stent malapposition remains a widely acknowledged problem in interventional cardiology. In 2012, a report from the International Working Group for Intravascular Optical Coherence Tomography Standardization and Validation was published, stating that OCT is more accurate for stent malapposition assessment, consequently being the preferred modality for reducing the risk of stent thrombosis [39]. In the following years, not only the OPUS-CLASS study, but also the ILUMIEN III: OPTIMIZE PCI trial displayed similar results with regard to acute malapposition imaging by OCT and IVUS. The same lesions were analyzed with both techniques and acute malapposition was detected two times more frequently by OCT than IVUS, i.e., 39% vs. 14% and 38.5% vs. 19.3%, in the OPUS-CLASS Study and ILUMIEN III: OPTIMIZE PCI trial, respectively [19,40]. Maehara et al. compared stent expansion in OCT- and IVUS-guided PCI from the ILUMIEN and ADAPT-DES studies, finding a higher prevalence of post-PCI stent malapposition detected by OCT, although the rate of major malapposition was similar after OCT- and IVUS-guided stenting [24]. However, if angiography suggests late acquired stent malapposition, IVUS is recommended because the evaluation of the entire vessel wall is possible, which is not the case for OCT [1]. The exhibited research clearly states that in terms of acute stent malapposition OCT is the superior modality related to IVUS. However, both modalities substantially contribute to procedure optimization, considering that each technique is able to detect stent malapposition and stent underexpansion—well-known predictors of adverse clinical outcomes. The current European consensus states that the minimal stent area (MSA) ≥80% in relation to vessel lumen area reference and/or a MSA > 4.5 mm^2^ in an OCT image is regarded acceptable and does not require additional invasive treatment [3].

Intravascular imaging modalities were also shown to be useful in coronary artery spasm (CAS) detection. IVUS can provide clues to differentiate vasospastic from nonvasospastic angina by allowing to identify small lesion site plaque burden, negative remodeling and diffuse coronary intimal thickening reflecting intimal hyperplasia in vasospastic angina. IVUS is also able to detect the presence of occult atherosclerotic lesions at the site of focal coronary artery spasm, even in the lack of angiographic signs of disease. [41] Nevertheless, OCT imaging can provide detailed information about the structural changes of coronary arteries in CAS patients. Studies have shown that in spastic-prone coronary arteries, an increased medial area and thickness can be found [42]. Furthermore, the presence of erosion at the coronary spasm site was identified in one-third of the cases, whereas the presence of luminal irregularities in two-thirds of cases [43]. OCT can also be useful in the assessment of thrombus at the spasm site [44].

Furthermore, OCT is more reliable than IVUS at identifying neoatherosclerosis, which is associated with very late stent restenosis or thrombosis [1]. Due to the different underlying mechanism of OCT, it is possible to evaluate tissue coverage of stent struts, and therefore, prevent stent thrombosis [41]. OCT seems especially suitable for assessing thrombus, not only due to the higher resolution, but also the visible attenuation of the OCT signal caused by red blood cells present inside the thrombus. It is described as an irregular mass (≥250 µm) protruding into the lumen [42]. OCT and IVUS are useful tools in PCI effect monitoring and predicting future major adverse cardiovascular events. OCT is the modality of choice for visualizing neoatherosclerosis, stent restenosis or stent thrombosis, due to the higher resolution of obtained images [3]. Several studies analyzed the usefulness of OCT and IVUS in assessment of tissue protrusion. In IVUS, it is imaged as a relatively high echogenic signal, while in OCT, it is observed as a smooth surface with high signal attenuation.

**Figure 4:**
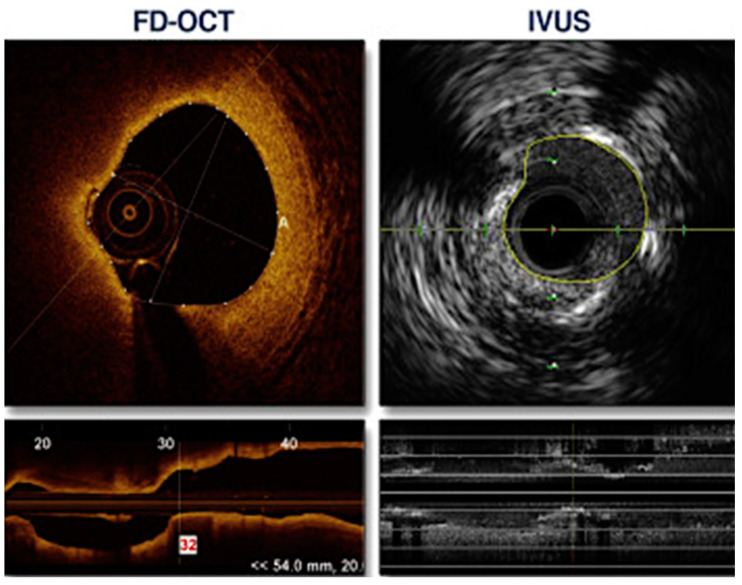
An example of a comparison between OCT- and IVUS-derived images and measurements of the same lesion in the circumflex coronary artery. The minimal lumen area was 2.75 mm^2^ in OCT and 3.50 mm^2^ by IVUS. Adapted with permission from [40]. Under an Elsevier User license, copyright year 2013.

Moreover, OCT can detect plaques prone to rupture. The advantage of OCT stems from its higher resolution in comparison to IVUS, which allows a more precise assessment [43] (Figure 4). Rui et al. analyzed 348 slices from the same lesions using both OCT and IVUS. The mean value difference between OCT and IVUS cap thickness measurements was 1.83% (*p* = 0.031). However, the mean value of point-to-point differences was 35.76%. The study group concluded that there were significant differences between IVUS and OCT plaque cap thickness measurements [44]. Similar conclusions were made by Ueki et al., who analyzed lesions with RF-IVUS and OCT. Out of the 208 lesions classified as TCFA (thin cap fibroatheroma) by RF-IVUS, 14 (6.7%) were also classified as TCFA in OCT, 60 (28.8%) as ThCFA (thick cap fibroatheroma), 101 (48.6%) as fibrosclerotic, 29 (13.9%) as fibrous and 4 (1.9%) as a normal vessel. All OCT assed TCFA (*n* = 14) were confirmed as TCFA by RF-IVUS. The concordance rate between RF-IVUS and OCT for TCFA diagnosis was 29.7% [45].

Kobayashi et al. sought to determine the differences in stent expansion between an OCT-guided and IVUS-guided rotational atherectomy. Burr upsizing was more frequent (55% vs. 32%, *p* = 0.001) and the final burr size was larger (1.75 [1.50–1.75] vs. 1.50 [1.50–1.75] mm, *p* < 0.001) in the OCT-guided group. The stent expansion percentage was greater in the OCT group (83 ± 15% vs. 72 ± 16%, *p* = 0.0004). The authors concluded that perhaps an OCT-guided rotational atherectomy may be the modality of choice when treating calcified coronary lesions [46].

The ILUMIEN III OPTIMIZE PCI trial examined clinical outcomes of an OCT- or IVUS-guided PCI. The results state that the assessment of non-complex lesions did not show a statistical difference in clinical outcomes at a 12-month follow-up between the two methods regarding target lesion failure (2.0% OCT; 3.7% IVUS) and major adverse cardiovascular events (9.8% OCT; 9.1% IVUS) [47]. Moreover, Kubo et al. conducted a study in which target vessel failure within 12 months after an OCT- or IVUS-guided PCI was the primary outcome. There was no statistically significant difference between the two modalities (5.2% OCT; 4.9% IVUS, *p* = 0.042 for non-inferiority testing [38]. Furthermore, Jones et al. analyzed in a cohort study of 87,166 patients the follow-up mortality in people who underwent an OCT- or IVUS-guided PCI. They suggest that the use of OCT and IVUS are of comparable value in terms of cardiovascular mortality [48]. Importantly, both modalities enable to optimize PCI. The studies using IVUS (ULTIMATE TRIAL and CLI-OPCI II TRIAL) showed that the suboptimal implantation of a stent significantly increases the risk of MACE at follow-up [49,50]. Therefore, PCI optimization with OCT and IVUS imaging improves patients’ outcomes.

A recent meta-analysis by Saleh et al. sought to compare the clinical outcomes between OCT-guided and IVUS-guided low-risk percutaneous coronary intervention [51]. The authors included 5 studies, with a total of 1544 patients. The analysis showed a similar risk of major cardiac adverse events (OCT 5.0% vs. IVUS 4.7%, *p* = 0.90), risk of all-cause death (OCT 2.7% vs. IVUS 1.7%, *p* = 0.44), myocardial infarction (OCT 1.5% vs. IVUS 1.3%, *p* = 0.76), stent thrombosis (OCT 0.3% vs. IVUS 0.4%, *p* = 0.66) and target lesion revascularization (OCT 2.2% vs. IVUS 2.6%, *p* = 0.58). These findings strongly indicate that OCT and IVUS represent comparable modalities for PCI guidance when it comes to clinical outcomes.

**Figure 5:**
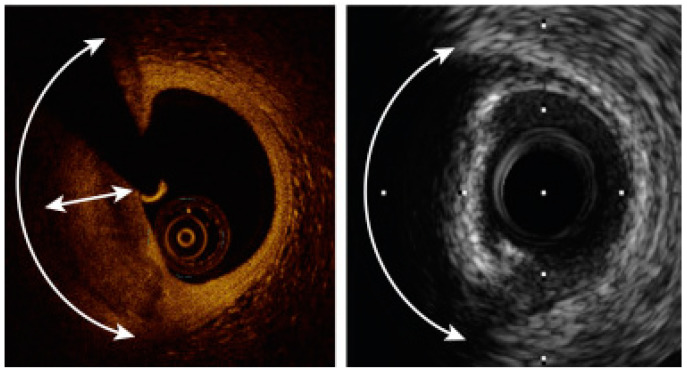
A comparison between the same calcified plaque. To the left an OCT image, to the right an IVUS image. The calcium angle (curved arrow) can be measured by both modalities and is 130°; however, calcium thickness (double-headed straight arrow) can only be measured by OCT. Adapted with permission from [1]. Under Elsevier User license, copyright year 2017.

In summary, in clinical practice, the beneficial impact of intravascular imaging on outcomes is related to each step of invasive management of coronary stenosis:

(i) More individualized lesion preparation based on the identified plaque morphology (i.e., OCT might be employed as a stent expansion tool since the presence of calcifications covering >180° of the vessel circumference and the length of these calcifications >5 mm in the OCT assessment increase the risk of stent underexpansion [30,52]) (Figure 5). In such patients, high-speed rotablation, orbital atherectomy or lithotripsy might be electively utilized to ensure successful PCI result [30,53,54,55].
(ii) Precise stent sizing and meticulous post dilatation ensuring adequate stent expansion and strut apposition, thereby decreasing the risk of complications [3,4,5,51].
(iii) In case of PCI failure, the critical role of OCT and IVUS has been well documented in the identification of the mechanisms underlying stent thrombosis and restenosis, thus facilitating their appropriate management [3,8,10]. Since the clinical adoption of the intravascular imaging modalities is still suboptimal in cathlabs across the world, of paramount importance is the ongoing post-graduate training of interventional cardiology community accompanied by sufficient reimbursement of these clinical outcome-improving techniques.

## 5. Combined IVUS OCT Catheters

An analysis of a single vessel by both IVUS and OCT requires the use of two separate catheters. Such a method is associated with some limitations, primarily an accurately overlapping thorough vessel analysis in images obtained by both modalities, as well as an increased risk of procedural side effects [56,57]. In order to address this matter, combined catheters were designed, which register the exact same area by both modalities at the same time. During the past decade, significant progress in this area has been made with the first of such a catheter used in the clinical settings in 2018. Such a system overlaps IVUS and OCT images, enabling a complete vessel wall visualization and evaluation [57]. Combining two techniques with each other within one catheter will deliver a wide range of available data, resulting in an optimization of the therapeutic approach (Table 3).

## 6. Conclusions

**Figure 6:**
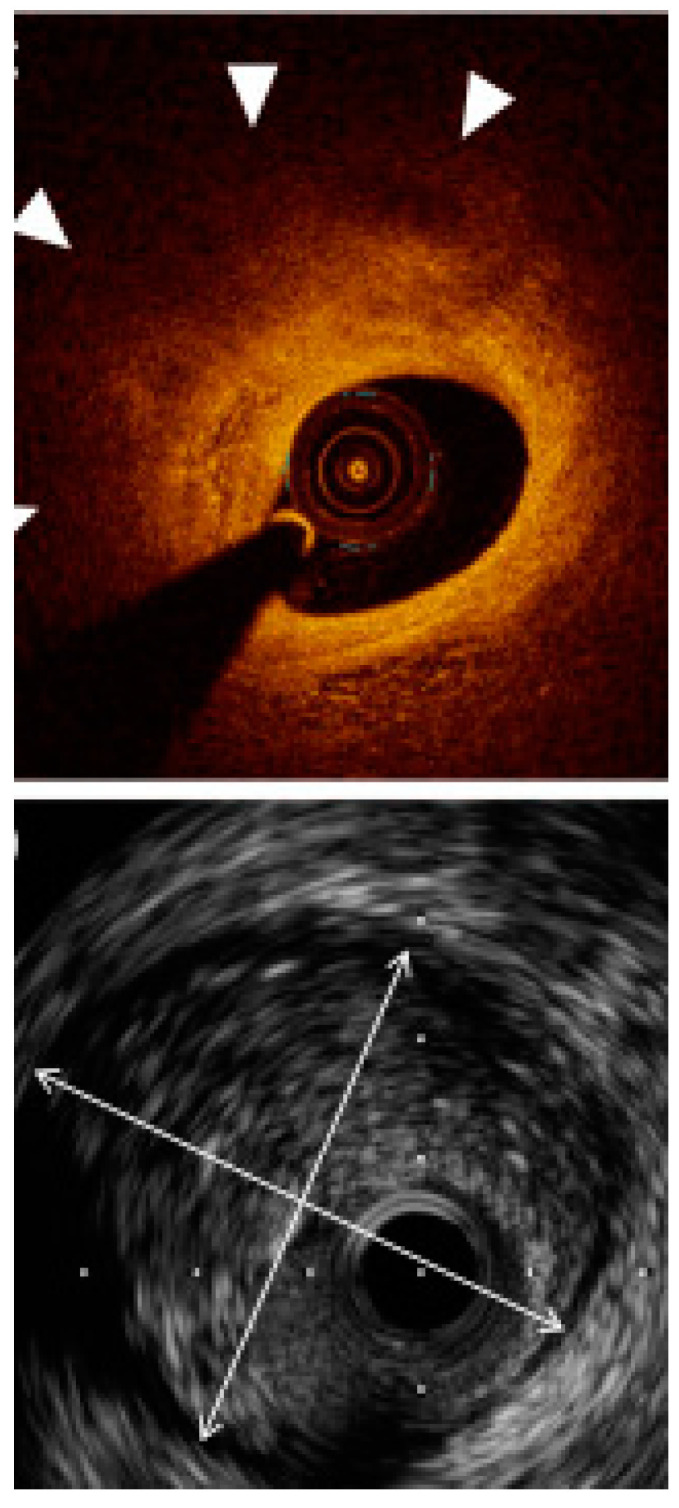
An example of vessel sizing in relation to external elastic lamina (EEL). IVUS (below) was able to show the full EEL diameter (arrow heads), whereas OCT did not visualize the EEL border due to the plaque attenuation and consequently lower signal penetration. Adapted with permission from [1]. Under Elsevier User license, copyright year 2017.

Not only clinical studies but also meta-analyses have demonstrated that both OCT and IVUS improve PCI results, reducing mortality, major adverse cardiovascular events, and the length of the hospitalization [4,6,48]. The benefits seem to be the greatest among the high-risk patients and complex lesions. In clinical practice, physicians should take into consideration the differences between the two methods, mainly higher resolution but lower penetration of OCT and, conversely, lower resolution but higher penetration of IVUS, in order to establish the optimal strategy (Figure 6). The presented studies demonstrate increased applicability in day-to-day practice; presumably, both modalities may be an integral element of routine cardiovascular imaging examinations in the future. All interventionalists should become familiar and regularly trained with the two modalities. Large-scale trials are underway to specifically evaluate the long-term prognostic impact of intravascular imaging use. Moreover, ongoing efforts are made to enhance new alterations of both modalities i.e., high resolution IVUS or OCT 3D reconstructions. Additionally, studies combining OCT and IVUS into a single catheter are in progress. These innovations are undoubtedly expected to bring further marked advancements in this field of cardiology.

## Figures and Tables

**Table 1:** Most important IVUS and OCT characteristics.

IVUS	Characteristic	OCT
Ultrasound	Type of wave	Infrared
5–6 mm	Tissue penetration depth	Up to 2.5 mm
Easily possible	Ability to visualize EEL	Very hard
Low	Resolution	High
No	Contrast usage	Yes
Possible	Ability of left main lesions assessment	Impossible
Medium	Repeatability of measurement	High

**Table 2:** A summary of recent studies comparing OCT vs. IVUS.

Study	Study Type	Aims of Investigation	Results
Ramasamy et al. [36] (*n* = 6919)	Meta-analysis	IVUS vs. OCT in detection of functionally significant intermediate non-left main coronary artery stenoses.	IVUS and OCT have similar sensitivity in predicting haemodynamically significant lesions (IVUS-MLA: 0.747 vs. OCT-MLA 0.732, *p* = 0.519). OCT-MLA had a higher specificity (0.763 vs. 0.665, *p* < 0.001) and diagnostic accuracy in detecting flow-limiting stenoses than IVUS-MLA (AUC 0.810 vs. 0.754, *p* = 0.045).
Habara et al. [37] (*n* = 70)	Randomized controlled trial	Evaluation of FD-OCT guidance for coronary stent implantation compared with IVUS guidance in patients with stable and unstable angina.	Smaller stent expansion in the FD-OCT guided stent implantation in comparison to the IVUS guided group (minimum and mean stent area, focal and diffuse stent expansion were smaller in the FD-OCT group, *p* < 0.05). Frequency of significant residual reference segment stenosis at the proximal edge was higher in the FD-OCT group (*p* < 0.05). No significant differences in pre-baloon dilatation and stent size.
OPINION Kubo et al. [38] (*n* = 829)	Randomized controlled trial	Comparison of OFDI-guided PCI compared with IVUS-guided PCI in terms of clinical outcomes.	12-month clinical outcome in patients undergoing OFDI-guided PCI was non-inferior to that of patients undergoing IVUS-guided PCI, defined by target vessel failure (composite of cardiac death, target-vessel related myocardial infarction, and ischaemia-driven target vessel revascularization). stent diameter was smaller in the OCT group (*p* = 0.005), with a tendency towards longer stents in OCT guided PCI (*p =* 0.06).
ILUMIEN III: OPTIMIZE PCI Ali, Maehara et al. [19] (*n* = 450)	Randomized controlled trial	Investigation of OCT and IVUS guided stent sizing in comparison with coronary angiography.	OCT guidance was non-inferior to IVUS guidance (one-sided 97.5% lower CI—0.70 mm^2^; *p* = 0.001), but also not superior (*p* = 0.42). OCT guidance was also found not to be superior to angiography guidance (*p* = 0.12). At a 12-month follow up there was no statistical difference in clinical outcomes between IVUS and OCT. Acute stent malapposition was detected two times more frequently by OCT than IVUS (38.5% vs. 19.3%).
OPUS-CLASS Kubo et al. [40] (*n* = 100)	Prospective study	Investigation of reliability of frequency domain optical coherence tomography (FD-OCT) for coronary measurements compared with quantitative coronary angiography (QCA) and intravascular ultrasound (IVUS).	The minimum lumen area measured byI VUS was significantly greater than that by FD-OCT (3.68 2.06 mm^2^ vs. 3.27 2.22 mm^2^, *p* < 0.001). Acute stent malapposition was detected two times more frequently by OCT than IVUS (39% vs. 14%).
Jones et al. [48] (*n* = 87,166)	Cohort study	Determination of the effect on long-term survival of patients who underwent an OCT- or an IVUS-guided PCI.	OCT-guided procedures were associated with greater procedural success rates and reduced in-hospital MACE rate. A significant difference in mortality was observed between patients who underwent OCT-guided PCI (7.7%) compared with patients who underwent either IVUS-guided (12.2%) or angiography-guided (15.7%; *p* < 0.0001) PCI Both intravascular modalities were predictors of survival, proving the superiority of clinical outcomes when the new imaging techniques were part of the diagnostic process
Saleh et al. [51] (*n* = 1544)	Meta-analysis	Comparison of the clinical outcomes between OCT-guided and IVUS-guided low risk percutaneous coronary intervention.	The analysis showed a similar risk of major cardiac adverse events (OCT 5.0% vs. IVUS 4.7%, *p* = 0.90), risk of all-cause death (OCT 2.7% vs. IVUS 1.7%, *p* = 0.44), myocardial infarction (OCT 1.5% vs. IVUS 1.3%, *p* = 0.76), stent thrombosis (OCT 0.3% vs. IVUS 0.4%, *p* = 0.66) and target lesion revascularization (OCT 2.2% vs. IVUS 2.6%, *p* = 0.59)

**Table 3:** Comparison of visualization and assessment capabilities between IVUS and OCT.

OCT	Visualization and Assessment of	IVUS
=	Non-complex lesions	=
	Left main assessment and stenting optimization	+
+	Acute stent malapposition	
+	Neoatherosclerosis	
+	Stent thrombosis	
+	Plaques prone to rupture	
+	Calcified plaques	

## Data Availability

All the abovementioned data can be found in the references.

## References

[1] Maehara A., Matsumura M., Ali Z.A., Mintz G.S., Stone G.W. (2017). IVUS-Guided Versus OCT-Guided Coronary Stent Implantation: A Critical Appraisal. JACC Cardiovasc. Imaging.

[2] Oosterveer T.T.M., van der Meer S.M., Scherptong R.W.C., Jukema J.W. (2020). Optical Coherence Tomography: Current Applications for the Assessment of Coronary Artery Disease and Guidance of Percutaneous Coronary Interventions. Cardiol. Ther..

[3] Ali Z.A., Karimi Galougahi K., Mintz G.S., Maehara A., Shlofmitz R.A., Mattesini A. (2021). Intracoronary optical coherence tomography: State of the art and future directions. EuroIntervention.

[4] Bartus S., Rzeszutko L., Januszek R. (2022). Optical coherence tomography enhanced by novel software to better visualize the mechanism of atherosclerosis and improve the effects of percutaneous coronary intervention. Kardiol. Pol..

[5] Pawlowski T., Legutko J., Kochman J., Roleder T., Pregowski J., Chmielak Z., Kubica J., Ochala A., Parma R., Grygier M. (2022). Clinical use of intracoronary imaging modalities in Poland. Expert opinion of the Association of Cardiovascular Interventions of the Polish Cardiac Society. Kardiol. Pol..

[6] Vallabhajosyula S., El Hajj S.C., Bell M.R., Prasad A., Lerman A., Rihal C.S., Holmes D.R., Barsness G.W. (2020). Intravascular ultrasound, optical coherence tomography, and fractional flow reserve use in acute myocardial infarction. Catheter. Cardiovasc. Interv..

[7] Alfonso F., Paulo M., Gonzalo N., Dutary J., Jimenez-Quevedo P., Lennie V., Escaned J., Banuelos C., Hernandez R., Macaya C. (2012). Diagnosis of spontaneous coronary artery dissection by optical coherence tomography. J. Am. Coll. Cardiol..

[8] Collet J.P., Thiele H., Barbato E., Barthelemy O., Bauersachs J., Bhatt D.L., Dendale P., Dorobantu M., Edvardsen T., Folliguet T. (2021). 2020 ESC Guidelines for the management of acute coronary syndromes in patients presenting without persistent ST-segment elevation. Eur. Heart J..

[9] Ibanez B., James S., Agewall S., Antunes M.J., Bucciarelli-Ducci C., Bueno H., Caforio A.L.P., Crea F., Goudevenos J.A., Halvorsen S. (2017). 2017 ESC Guidelines for the management of acute myocardial infarction in patients presenting with ST-segment elevation. Rev. Esp. Cardiol..

[10] Johnson T.W., Raber L., di Mario C., Bourantas C., Jia H., Mattesini A., Gonzalo N., de la Torre Hernandez J.M., Prati F., Koskinas K. (2019). Clinical use of intracoronary imaging. Part 2: Acute coronary syndromes, ambiguous coronary angiography findings, and guiding interventional decision-making: An expert consensus document of the European Association of Percutaneous Cardiovascular Interventions. Eur. Heart J..

[11] de la Torre Hernandez J.M., Hernandez Hernandez F., Alfonso F., Rumoroso J.R., Lopez-Palop R., Sadaba M., Carrillo P., Rondan J., Lozano I., Ruiz Nodar J.M. (2011). Prospective application of pre-defined intravascular ultrasound criteria for assessment of intermediate left main coronary artery lesions results from the multicenter LITRO study. J. Am. Coll. Cardiol..

[12] Neumann F.J., Sousa-Uva M., Ahlsson A., Alfonso F., Banning A.P., Benedetto U., Byrne R.A., Collet J.P., Falk V., Head S.J. (2019). 2018 ESC/EACTS Guidelines on myocardial revascularization. Eur. Heart J..

[13] Yock P.G., Linker D.T., Angelsen B.A. (1989). Two-dimensional intravascular ultrasound: Technical development and initial clinical experience. J. Am. Soc. Echocardiogr..

[14] Sung J.H., Jeong J.S. (2018). Development of High-Frequency (>60 MHz) Intravascular Ultrasound (IVUS) Transducer by Using Asymmetric Electrodes for Improved Beam Profile. Sensors.

[15] Garcia-Garcia H.M., Gogas B.D., Serruys P.W., Bruining N. (2011). IVUS-based imaging modalities for tissue characterization: Similarities and differences. Int. J. Cardiovasc. Imaging.

[16] Shlofmitz E., Jeremias A., Parviz Y., Karimi Galougahi K., Redfors B., Petrossian G., Edens M., Matsumura M., Maehara A., Mintz G.S. (2021). External elastic lamina vs. luminal diameter measurement for determining stent diameter by optical coherence tomography: An ILUMIEN III substudy. Eur. Heart J. Cardiovasc. Imaging.

[17] Ono M., Kawashima H., Hara H., Gao C., Wang R., Kogame N., Takahashi K., Chichareon P., Modolo R., Tomaniak M. (2020). Advances in IVUS/OCT and Future Clinical Perspective of Novel Hybrid Catheter System in Coronary Imaging. Front. Cardiovasc. Med..

[18] Serruys P.W., Katagiri Y., Sotomi Y., Zeng Y., Chevalier B., van der Schaaf R.J., Baumbach A., Smits P., van Mieghem N.M., Bartorelli A. (2017). Arterial Remodeling After Bioresorbable Scaffolds and Metallic Stents. J. Am. Coll. Cardiol..

[19] Ali Z.A., Maehara A., Genereux P., Shlofmitz R.A., Fabbiocchi F., Nazif T.M., Guagliumi G., Meraj P.M., Alfonso F., Samady H. (2016). Optical coherence tomography compared with intravascular ultrasound and with angiography to guide coronary stent implantation (ILUMIEN III: OPTIMIZE PCI): A randomised controlled trial. Lancet.

[20] Nair A., Kuban B.D., Tuzcu E.M., Schoenhagen P., Nissen S.E., Vince D.G. (2002). Coronary plaque classification with intravascular ultrasound radiofrequency data analysis. Circulation.

[21] Bourantas C.V., Garcia-Garcia H.M., Farooq V., Maehara A., Xu K., Genereux P., Diletti R., Muramatsu T., Fahy M., Weisz G. (2013). Clinical and angiographic characteristics of patients likely to have vulnerable plaques: Analysis from the PROSPECT study. JACC Cardiovasc. Imaging.

[22] Mariani J., Guedes C., Soares P., Zalc S., Campos C.M., Lopes A.C., Spadaro A.G., Perin M.A., Filho A.E., Takimura C.K. (2014). Intravascular ultrasound guidance to minimize the use of iodine contrast in percutaneous coronary intervention: The MOZART (Minimizing cOntrast utiliZation With IVUS Guidance in coRonary angioplasTy) randomized controlled trial. JACC Cardiovasc. Interv..

[23] Mintz G.S., Popma J.J., Pichard A.D., Kent K.M., Satler L.F., Chuang Y.C., Ditrano C.J., Leon M.B. (1995). Patterns of calcification in coronary artery disease. A statistical analysis of intravascular ultrasound and coronary angiography in 1155 lesions. Circulation.

[24] Maehara A., Ben-Yehuda O., Ali Z., Wijns W., Bezerra H.G., Shite J., Genereux P., Nichols M., Jenkins P., Witzenbichler B. (2015). Comparison of Stent Expansion Guided by Optical Coherence Tomography Versus Intravascular Ultrasound: The ILUMIEN II Study (Observational Study of Optical Coherence Tomography [OCT] in Patients Undergoing Fractional Flow Reserve [FFR] and Percutaneous Coronary Intervention). JACC Cardiovasc. Interv..

[25] Erlinge D., Maehara A., Ben-Yehuda O., Botker H.E., Maeng M., Kjoller-Hansen L., Engstrom T., Matsumura M., Crowley A., Dressler O. (2021). Identification of vulnerable plaques and patients by intracoronary near-infrared spectroscopy and ultrasound (PROSPECT II): A prospective natural history study. Lancet.

[26] Tomaniak M., Hartman E.M.J., Tovar Forero M.N., Wilschut J., Zijlstra F., Van Mieghem N.M., Kardys I., Wentzel J., Daemen J. (2022). Near-infrared spectroscopy to predict plaque progression in plaque-free artery regions. EuroIntervention.

[27] Zhang J., Gao X., Kan J., Ge Z., Han L., Lu S., Tian N., Lin S., Lu Q., Wu X. (2018). Intravascular Ultrasound Versus Angiography-Guided Drug-Eluting Stent Implantation: The ULTIMATE Trial. J. Am. Coll. Cardiol..

[28] Ochijewicz D., Tomaniak M., Koltowski L., Rdzanek A., Pietrasik A., Kochman J. (2017). Intravascular imaging of coronary artery disease: Recent progress and future directions. J. Cardiovasc. Med..

[29] Kedhi E., Berta B., Roleder T., Hermanides R.S., Fabris E., AJJ I.J., Kauer F., Alfonso F., von Birgelen C., Escaned J. (2021). Thin-cap fibroatheroma predicts clinical events in diabetic patients with normal fractional flow reserve: The COMBINE OCT-FFR trial. Eur. Heart J..

[30] Yabushita H., Bouma B.E., Houser S.L., Aretz H.T., Jang I.K., Schlendorf K.H., Kauffman C.R., Shishkov M., Kang D.H., Halpern E.F. (2002). Characterization of human atherosclerosis by optical coherence tomography. Circulation.

[31] Tomaniak M., Katagiri Y., Modolo R., de Silva R., Khamis R.Y., Bourantas C.V., Torii R., Wentzel J.J., Gijsen F.J., van Soest G. (2020). Vulnerable plaques and patients: State-of-the-art. Eur. J..

[32] Fujino A., Mintz G.S., Matsumura M., Lee T., Kim S.Y., Hoshino M., Usui E., Yonetsu T., Haag E.S., Shlofmitz R.A. (2018). A new optical coherence tomography-based calcium scoring system to predict stent underexpansion. EuroIntervention.

[33] Li J., Li X., Mohar D., Raney A., Jing J., Zhang J., Johnston A., Liang S., Ma T., Shung K.K. (2014). Integrated IVUS-OCT for real-time imaging of coronary atherosclerosis. JACC Cardiovasc. Imaging.

[34] Ota H., Kawase Y., Kondo H., Miyake T., Kamikawa S., Okubo M., Tsuchiya K., Matsuo H., Honye J., Ueno K. (2013). A case report of acute myocardial infarction induced by coronary spasm. Intravascular findings. Int. Heart J..

[35] Tanaka A., Shimada K., Tearney G.J., Kitabata H., Taguchi H., Fukuda S., Kashiwagi M., Kubo T., Takarada S., Hirata K. (2011). Conformational change in coronary artery structure assessed by optical coherence tomography in patients with vasospastic angina. J. Am. Coll. Cardiol..

[36] Ramasamy A., Chen Y., Zanchin T., Jones D.A., Rathod K., Jin C., Onuma Y., Zhang Y.J., Amersey R., Westwood M. (2020). Optical coherence tomography enables more accurate detection of functionally significant intermediate non-left main coronary artery stenoses than intravascular ultrasound: A meta-analysis of 6919 patients and 7537 lesions. Int. J. Cardiol..

[37] Habara M., Nasu K., Terashima M., Kaneda H., Yokota D., Ko E., Ito T., Kurita T., Tanaka N., Kimura M. (2012). Impact of frequency-domain optical coherence tomography guidance for optimal coronary stent implantation in comparison with intravascular ultrasound guidance. Circ. Cardiovasc. Interv..

[38] Kubo T., Shinke T., Okamura T., Hibi K., Nakazawa G., Morino Y., Shite J., Fusazaki T., Otake H., Kozuma K. (2017). Optical frequency domain imaging vs. intravascular ultrasound in percutaneous coronary intervention (OPINION trial): One-year angiographic and clinical results. Eur. Heart J..

[39] Tearney G.J., Regar E., Akasaka T., Adriaenssens T., Barlis P., Bezerra H.G., Bouma B., Bruining N., Cho J.M., Chowdhary S. (2012). Consensus standards for acquisition, measurement, and reporting of intravascular optical coherence tomography studies: A report from the International Working Group for Intravascular Optical Coherence Tomography Standardization and Validation. J. Am. Coll. Cardiol..

[40] Kubo T., Akasaka T., Shite J., Suzuki T., Uemura S., Yu B., Kozuma K., Kitabata H., Shinke T., Habara M. (2013). OCT compared with IVUS in a coronary lesion assessment: The OPUS-CLASS study. JACC Cardiovasc. Imaging.

[41] Won H., Shin D.H., Kim B.K., Mintz G.S., Kim J.S., Ko Y.G., Choi D., Jang Y., Hong M.K. (2013). Optical coherence tomography derived cut-off value of uncovered stent struts to predict adverse clinical outcomes after drug-eluting stent implantation. Int. J. Cardiovasc. Imaging.

[42] Kume T., Akasaka T., Kawamoto T., Ogasawara Y., Watanabe N., Toyota E., Neishi Y., Sukmawan R., Sadahira Y., Yoshida K. (2006). Assessment of coronary arterial thrombus by optical coherence tomography. Am. J. Cardiol..

[43] Roleder T., Jakala J., Kaluza G.L., Partyka L., Proniewska K., Pociask E., Zasada W., Wojakowski W., Gasior Z., Dudek D. (2015). The basics of intravascular optical coherence tomography. Postepy Kardiol. Interwencyjnej..

[44] Lv R., Maehara A., Matsumura M., Wang L., Wang Q., Zhang C., Guo X., Samady H., Giddens D.P., Zheng J. (2020). Using optical coherence tomography and intravascular ultrasound imaging to quantify coronary plaque cap thickness and vulnerability: A pilot study. Biomed. Eng. Online.

[45] Ueki Y., Yamaji K., Losdat S., Karagiannis A., Taniwaki M., Roffi M., Otsuka T., Koskinas K.C., Holmvang L., Maldonado R. (2021). Discordance in the diagnostic assessment of vulnerable plaques between radiofrequency intravascular ultrasound versus optical coherence tomography among patients with acute myocardial infarction: Insights from the IBIS-4 study. Int. J. Cardiovasc. Imaging.

[46] Kobayashi N., Ito Y., Yamawaki M., Araki M., Obokata M., Sakamoto Y., Mori S., Tsutsumi M., Honda Y., Makino K. (2020). Optical coherence tomography-guided versus intravascular ultrasound-guided rotational atherectomy in patients with calcified coronary lesions. EuroIntervention.

[47] Ali Z.A., Karimi Galougahi K., Maehara A., Shlofmitz R.A., Fabbiocchi F., Guagliumi G., Alfonso F., Akasaka T., Matsumura M., Mintz G.S. (2021). Outcomes of optical coherence tomography compared with intravascular ultrasound and with angiography to guide coronary stent implantation: One-year results from the ILUMIEN III: OPTIMIZE PCI trial. EuroIntervention.

[48] Jones D.A., Rathod K.S., Koganti S., Hamshere S., Astroulakis Z., Lim P., Sirker A., O’Mahony C., Jain A.K., Knight C.J. (2018). Angiography Alone Versus Angiography Plus Optical Coherence Tomography to Guide Percutaneous Coronary Intervention: Outcomes From the Pan-London PCI Cohort. JACC Cardiovasc. Interv..

[49] Gao X.F., Ge Z., Kong X.Q., Kan J., Han L., Lu S., Tian N.L., Lin S., Lu Q.H., Wang X.Y. (2021). 3-Year Outcomes of the ULTIMATE Trial Comparing Intravascular Ultrasound Versus Angiography-Guided Drug-Eluting Stent Implantation. JACC Cardiovasc. Interv..

[50] Prati F., Romagnoli E., Burzotta F., Limbruno U., Gatto L., La Manna A., Versaci F., Marco V., Di Vito L., Imola F. (2015). Clinical Impact of OCT Findings During PCI: The CLI-OPCI II Study. JACC Cardiovasc. Imaging.

[51] Saleh Y., Al-Abcha A., Abdelkarim O., Abdelfattah O.M., Abela G.S., Hashim H., Goel S.S., Kleiman N.S. (2022). Meta-Analysis Investigating the Role of Optical Coherence Tomography Versus Intravascular Ultrasound in Low-Risk Percutaneous Coronary Intervention. Am. J. Cardiol..

[52] Hoffmann R., Mintz G.S., Popma J.J., Satler L.F., Kent K.M., Pichard A.D., Leon M.B. (1998). Treatment of calcified coronary lesions with Palmaz-Schatz stents. An intravascular ultrasound study. Eur. Heart J..

[53] Yamamoto M.H., Maehara A., Karimi Galougahi K., Mintz G.S., Parviz Y., Kim S.S., Koyama K., Amemiya K., Kim S.Y., Ishida M. (2017). Mechanisms of Orbital Versus Rotational Atherectomy Plaque Modification in Severely Calcified Lesions Assessed by Optical Coherence Tomography. JACC Cardiovasc. Interv..

[54] Ali Z.A., Brinton T.J., Hill J.M., Maehara A., Matsumura M., Karimi Galougahi K., Illindala U., Gotberg M., Whitbourn R., Van Mieghem N. (2017). Optical Coherence Tomography Characterization of Coronary Lithoplasty for Treatment of Calcified Lesions: First Description. JACC Cardiovasc. Imaging.

[55] Wanha W., Tomaniak M., Wanczura P., Bil J., Januszek R., Wolny R., Opolski M.P., Kuzma L., Janas A., Figatowski T. (2022). Intravascular Lithotripsy for the Treatment of Stent Underexpansion: The Multicenter IVL-DRAGON Registry. J. Clin. Med..

[56] Räber L., Heo J.H., Radu M.D., Garcia-Garcia H.M., Stefanini G.G., Moschovitis A., Dijkstra J., Kelbaek H., Windecker S., Serruys P.W. (2012). Offline fusion of co-registered intravascular ultrasound and frequency domain optical coherence tomography images for the analysis of human atherosclerotic plaques. EuroIntervention.

[57] Zeng Y., Tateishi H., Cavalcante R., Tenekecioglu E., Suwannasom P., Sotomi Y., Collet C., Nie S., Jonker H., Dijkstra J. (2017). Serial Assessment of Tissue Precursors and Progression of Coronary Calcification Analyzed by Fusion of IVUS and OCT: 5-Year Follow-Up of Scaffolded and Nonscaffolded Arteries. JACC Cardiovasc. Imaging.

